# Effectiveness and cost-effectiveness of rehabilitation after lumbar disc surgery (REALISE): design of a randomised controlled trial

**DOI:** 10.1186/1471-2474-14-124

**Published:** 2013-04-05

**Authors:** Teddy Oosterhuis, Maurits van Tulder, Wilco Peul, Judith Bosmans, Carmen Vleggeert-Lankamp, Lidwien Smakman, Mark Arts, Raymond Ostelo

**Affiliations:** 1 Department of Health Sciences, Faculty of Earth and Life Science, VU University Amsterdam and the EMGO Institute for Health and Care Research, Amsterdam, The Netherlands; 2 Department of Epidemiology and Biostatistics, VU University Medical Center, Amsterdam and the EMGO Institute for Health and Care Research, Amsterdam, The Netherlands; 3 Department of Neurosurgery, Leiden University Medical Center, Leiden, The Netherlands; 4 Department of Neurosurgery, Medical Center Haaglanden, The Hague, The Netherlands

**Keywords:** Rehabilitation, Exercise, Physiotherapy, Lumbar disc surgery, Lumbar disc herniation, Randomised controlled trial, Economic evaluation

## Abstract

**Background:**

Patients who undergo lumbar disc surgery for herniated discs, are advocated two different postoperative management strategies: a watchful waiting policy, or referral for rehabilitation immediately after discharge from the hospital. A direct comparison of the effectiveness and cost-effectiveness of these two strategies is lacking.

**Methods/Design:**

A randomised controlled trial will be conducted with an economic evaluation alongside to assess the (cost-) effectiveness of rehabilitation after lumbar disc surgery. Two hundred patients aged 18–70 years with a clear indication for lumbar disc surgery of a single level herniated disc will be recruited and randomly assigned to either a watchful waiting policy for first six weeks or exercise therapy starting immediately after discharge from the hospital. Exercise therapy will focus on resumption of activities of daily living and return to work. Therapists will tailor the intervention to the individual patient’s needs. All patients will be followed up by the neurosurgeon six weeks postoperatively. Main outcome measures are: functional status, pain intensity and global perceived recovery. Questionnaires will be completed preoperatively and at 3, 6, 9, 12 and 26 weeks after surgery. Data will be analysed according to the intention-to-treat principle, using a linear mixed model for continuous outcomes and a generalised mixed model for dichotomous outcomes. The economic evaluation will be performed from a societal perspective.

**Discussion:**

The results of this trial may lead to a more consistent postoperative strategy for patients who will undergo lumbar disc surgery.

**Trial registration:**

Netherlands Trial Register: NTR3156

## Background

The lumbosacral radicular syndrome, also called sciatica, is commonly caused by a herniated disc [1]. The syndrome is z=s: characterised by lower limb pain radiating below the knee in an area of the leg served by one or more lumbosacral nerve roots. Sometimes there are neurological phenomena such as sensory and motor deficits. In the Netherlands, the incidence of sciatica increased from 75,000 to 85,000 cases per year over the past decade [2,3]. The direct and indirect costs of patients suffering from sciatica approximate 1.2 billion Euros per year [2]. The natural course of sciatica is favourable in the majority of patients [4]. The international consensus is that surgical treatment is offered if the radiating leg pain persists despite a period of conservative management [5]. It is estimated that about 12,000 operations for herniated lumbar discs are performed in the Netherlands each year [3]. Rates of spinal surgery differ across countries and within one country [6]. Rates in the United States are 30% higher than in the Netherlands, 50–60% higher than in Canada and 80% higher than in the UK [7].

In the long term, surgical and non-surgical management are equally successful [8,9]. Recovery rates between 78 and 95% were found 1–2 years postoperatively [8-13] However, shortly after surgery results vary, with recovery rates of 46–75% 6–8 weeks postoperatively [8,9,13] This variation may be due to differences in defining recovery, but also due to different referral patterns for rehabilitation. It implies a poor outcome in up to 54% of the patients at short term follow up, and up to 22% of the patients at ≥ 1 year follow up. As this group of patients highly contributes to the direct and indirect costs of lumbar disc surgery, an important aim of postoperative treatment is to prevent the development of chronic symptoms [14].

Currently, postoperative care and management, including referral for rehabilitation (i.e. exercise therapy and advice provided by physiotherapists or exercise therapists), varies between hospitals and surgeons. A national survey in the Netherlands amongst spinal surgeons (both neurosurgeons and orthopaedic surgeons) showed that 65% of the surgeons always refer patients for postoperative physiotherapy after discharge, whereas 24% never refer patients for postoperative physiotherapy and 11% sometimes. Likewise, 45% of the surgeons consider physiotherapy after discharge to be essential, but 30% of the surgeons strongly disagree that physiotherapy would be essential [15]. A similar trend was seen in a national survey in the UK where 55% of the surgeons did not send their patients for physiotherapy following spinal surgery [16]. In another UK survey physiotherapists providing care to patients who had undergone lumbar disc surgery reported a wide variety of treatment contents being delivered. Moreover, this study revealed considerable variation in access to postoperative physiotherapy for outpatients [17].

Patients are usually referred for rehabilitation during hospitalisation [15]. The two most common options for management after discharge are continued rehabilitation or a watchful waiting policy. The first option consists of continued rehabilitation offered to all patients. The second option comprises watchful waiting during the first weeks advising to return to an active lifestyle, with postoperative treatment only for those patients having persisting symptoms after six to eight weeks. Several randomised controlled trials investigated the effectiveness of rehabilitation following primary lumbar disc surgery [18]. For exercise programmes starting 4–6 weeks postoperatively, there is moderate evidence that they are more effective in improving physical function, and low quality evidence that they are more effective than no treatment in decreasing pain. There is moderate evidence that high intensity exercises are more effective in improving physical function compared to low intensity exercises, and low quality evidence that they are more effective in decreasing pain. However, high quality studies assessing the effectiveness of immediate postoperative interventions are lacking [18]. Besides, data on cost-effectiveness of postoperative management is scarce [19]. Therefore, the aim of the Rehabilitation After Lumbar Disc Surgery (REALISE) study is to evaluate the effectiveness of an exercise therapy intervention starting in the first postoperative week, compared to a watchful waiting policy during the first 6–8 weeks after surgery. Furthermore, cost-effectiveness will be assessed by means of an economic evaluation from a societal perspective.

## Methods/Design

### Design

A multicentre randomised controlled trial will be conducted, together with a full economic evaluation.

### Setting

Participants will be recruited from 10 hospitals, both urban and regional, in three regions in the Netherlands, where lumbar disc surgery is performed. Primary care physiotherapists and exercise therapists in the catchment areas of these hospitals will provide rehabilitation following this lumbar disc surgery. Prior to the start of the trial all participating therapists will follow a training session in which both the study design and treatment protocol for the intervention group will be explained.

### Ethical approval

The Medical Ethics Review Board of the VU University Medical Centre approved the study protocol in September 2011 (registration number NL35897.029.11). Subsequently, local review boards of each participating hospital approved the protocol.

### Participants

Patients will be eligible for inclusion if they meet all of the following inclusion criteria:

lumbar disc herniation confirmed by MRI and physical signs of nerve root compression corresponding to the level of disc herniation,

between 18 and 70 years of age,

able to fill out Dutch questionnaires themselves,

and provide written informed consent.

Patients will be excluded if they meet any of the following criteria:

cauda equina syndrome,

neurogenic claudication,

co-morbidities of the lumbar spine (e.g., fractures, carcinomas, osteoporosis),

prior spinal surgery in the last 12 months,

previous lumbar disc surgery at the same level and same side,

pregnancy,

or contra-indications for exercise therapy (e.g., acute respiratory or cardiovascular complaints, acute systemic infections).

### Participant recruitment

**Figure 1:**
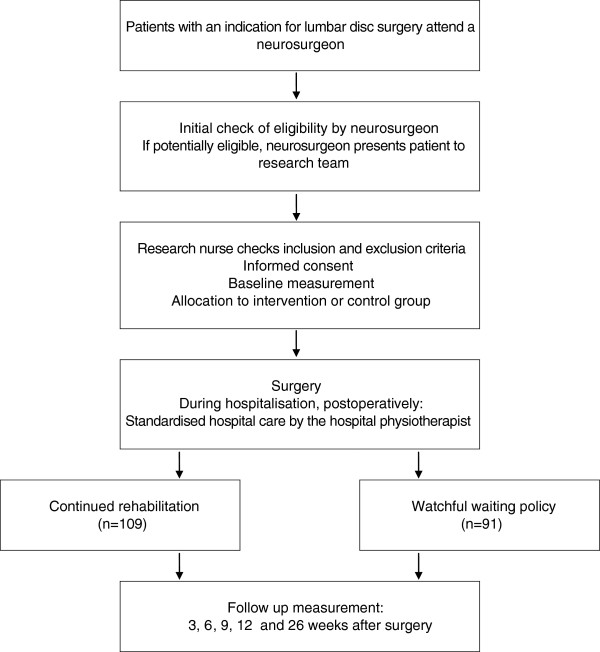
Flow diagram of the REALISE trial.

Figure 1 comprises the flow diagram of the REALISE trial. Neurosurgeons will inform all patients with a clear indication for lumbar disc surgery about the study and will refer potentially eligible participants to the research team prior to surgery. The neurosurgeon will register whether the patient would normally have been referred for postoperative rehabilitation or not. A research nurse will subsequently invite the patients for an intake preceding surgery and then check eligibility criteria and provide details about the trial procedures. If the patient consents to participate, baseline measurements will be administered.

### Treatment allocation

To conceal treatment allocation, randomisation lists per hospital will be generated by computer prior to study commencement by an independent person. To achieve the predetermined size of treatment and control group, weighted block randomisation (blocks of four) will be used. Directly after having received the completed baseline questionnaire, the research nurse will open the next consecutive a priori prepared numbered opaque envelope containing the assigned postoperative strategy. Patients allocated to the intervention group will receive a list of the participating therapists and are asked to make an appointment for the first treatment session in the first week after surgery. Within one week post-surgery the research nurse will contact the patient to ensure that an appointment has been made and to record the date of the first appointment.

### Intervention

During hospitalisation all participants, regardless of treatment allocation, will receive usual hospital care. This treatment, provided by a physiotherapist or nurse, mainly consists of providing advice and instructions for transfers and performing activities of daily living, in preparation for discharge. At discharge patients will receive a booklet providing advice and suggestions for exercises. These booklets are part of usual inpatient care and are provided by the participating hospitals.

#### Intervention group: continued rehabilitation

Participants in the intervention group will receive a referral for post-surgery exercise therapy in primary care starting the first week after discharge. During a six to eight week period, participants will receive one or two exercise therapy sessions per week, conform a treatment protocol designed for this trial including information and advice about rehabilitation. The six to eight week period reflects the period before patients consult their neurosurgeon again after the surgery. The exact duration of this period depends on the organisation in the participating hospital in which the patient was operated. The protocol is based on existing protocols for primary care exercise therapy, which were derived from a national clinical guideline [20]. The main goal is to gradually extend activities of daily living from personal care to housekeeping tasks in the short term and return to work and prepare for sports and leisure activities in the long term. In the first week, therapists will perform physical examinations and focus treatment on the ability and possibility to execute personal care activities and perform transfers (e.g., bed to stand, chair to stand) in the home situation. From the second week onward, exercises will be taught with gradually increasing intensity targeting limitations that were found in the initial postoperative assessment. The exact type of exercises is left to the therapists’ discretion, based on the outcomes of the physical examination and taking patients’ preferences into account. Therapists will provide tailored advice on lifestyle and the execution of activities of daily living. Exercises will aim to prepare for and support the resumption of daily activities. Treatment can be terminated before the end of the six to eight week period if the patient is fully recovered.

Per treatment session, participating therapists will fill out a registration form including amongst others, treatment goals on both a global and more specific level, whether homework was prescribed or not and, if applicable, the reason for terminating the treatment.

#### Control group: watchful waiting policy

Participants assigned to the control group will not receive exercise therapy immediately after discharge from hospital. Patients may consult their neurosurgeon or general practitioner in case of recurring or increasing complaints, but no exercise therapy or other allied health care intervention will be initiated. The research nurses will limit the extent to which they will provide advice in case they will be called by patients allocated to the control group. To prevent diminishing contrast between groups, only advice that has been given during the clinical phase will be repeated.

#### Follow-up consult neurosurgeon

Six to eight weeks after discharge a follow-up consult with the neurosurgeon will take place. Whether patients are referred for further treatment after this follow up consultation is left to the neurosurgeons’ discretion. We will measure all health care consumption using cost questionnaires (see section on Economic evaluation below).

### Outcomes

Table 1 provides an overview of the data collected during the trial. Baseline assessments will take place several days to several hours preoperatively, depending on usual logistic procedures in the participating hospitals. Follow-up measurements will take place at three days and 3, 6, 9, 12 and 26 weeks postoperatively. The study will use standardised instruments with demonstrated validity, reliability and responsiveness. Outcomes will be measured using online questionnaires, but postal questionnaires are available if requested. The baseline measures will include demographic data (such as age, gender and education), relevant prognostic factors and both primary and secondary outcomes.

**Table 1:** Overview of the data collection

**Outcome measures**		**Follow-up**						
	**Baseline**	**3 days**	**1 wk**	**3 wk**	**6 wk**	**9 wk**	**12 wk**	**26 wk**
Demographic data	X							
***Primary outcomes***								
Functional status (ODI)	X			X	X	X	X	X
Pain intensity leg (NRS)	X	X		X	X	X	X	X
Pain intensity back (NRS)	X	X		X	X	X	X	X
Global perceived recovery (GPE)				X	X	X	X	X
General health status (SF-12)	X			X	X	X	X	X
Health related quality of life (EQ-5D)	X			X	X	X	X	X
***Secondary outcomes***								
Psychosocial status (ÖMPSQ)	X							
Fear avoidance beliefs (FABQ)	X							
Expectations (CEQ)	X		X					
Pain Coping (PCI)	X							
***Prognostic factors***								
Length/severity of complaints	X							
Surgical complications		X						
***Economic evaluation***								
Absence from work (PRODISQ)					X		X	X
Cost questionnaire					X		X	X

ODI: Oswestry Disability Index; NRS: Numerical Rating Scale; GPE: Global Perceived Effect Scale; SF-12: Medical Outcome Study Short Form 12; EQ-D5: EuroQol; ÖMPSQ: Örebro Musculoskeletal Pain Screening Questionnaire; FABQ: Fear-Avoidance Beliefs Questionnaire; CEQ: credibility/expectancy questionnaire; PCI: Pain Coping Inventory; PRODISQ: Productivity and Disease Questionnaire; wk: week or weeks.

#### Prognostic factors

Prognostic factors indicating unfavourable outcome after lumbar disc surgery include length and severity of complaints preceding surgery and psychosocial factors. Complications during surgery are another prognostic factor which will be retrieved from the patient files during the 6-week assessment [21].

#### Primary outcome measures

The recommended core set of outcomes for low back pain research [22] will be used, and all measures are self-reported.

Functional status will be assessed by the Oswestry Disability Index (ODI) [23,24]. The ODI consists of 10 questions assessing aspects of daily living, each being scored on a six-point scale, ranging from 0–5. The total score comprises the 10 item-scores and ranges from 0 (no difficulty) to 50 (maximal difficulty).

Pain intensity will be measured for leg pain and low back pain on an 11-point numerical rating scale (0 = no pain to 10 = worst imaginable pain) [25,26]. Average pain intensity over the preceding week will be measured, for leg pain and low back pain separately. At three days postoperative the pain at the time of measurement will be assessed, again for leg and back pain separately.

Global perceived recovery will be evaluated with the seven-point Global Perceived Effect scale (GPE), ranging from “completely recovered” to “worse than ever” [22]. This will be dichotomised into success (completely and much recovered) and non-success (slightly recovered, no change, slightly worse, much worse and worse than ever).

The Medical Outcome Study Short Form 12 (SF-12) will be used to assess general health status [27,28]. The 12 items cover the dimensions physical function and mental health. Sum-scales can be derived with scores ranging from 0–100 per dimension. Higher scores reflect better health.

The EuroQol (EQ-D5) will be administered to assess health related quality of life [29,30]. This instrument evaluates 5 dimensions of quality of life on a three-point scale (no problems, moderate problems and severe problems). Utilities based on the EQ-5D will be estimated using the Dutch valuation tariff [31].

Costs will be measured from a societal perspective using cost questionnaires. Only costs related to leg and back pain are considered and will include costs of continued rehabilitation, other health care costs, patient and family costs, and missed days of unpaid work. The cost questionnaires and the therapists’ registrations of sessions completed are also used to check compliance to the allocated treatment and possible cross-over, i.e. receiving exercise therapy in the control group either by means of direct access or referral.

A modified version of the Productivity and Disease Questionnaire (PRODISQ) will be used to evaluate absence from paid work [32]. Dutch standard costs will be used to value resource utilisation [33]. Lost productivity costs will be estimated according to the friction cost approach [34] and the human capital approach.

#### Secondary outcome measures

Expectations of treatment outcome will be measured using the credibility/expectancy questionnaire (CEQ) [35,36]. The CEQ contains three nine-point credibility scales, two expectancy scales (0–100%) and one nine-point credibility item. Higher scores reflect more positive expectations. At baseline, expectations of surgery outcome will be elicited. Furthermore, to assess expectations regarding continued rehabilitation post-surgery and the watchful waiting policy, respectively, the reformulated CEQ item on expected success of treatment will be used. All CEQ baseline measures will be taken prior to treatment allocation. The CEQ will be used to measure expectations regarding the allocated strategy post-surgery one week postoperatively in the watchful waiting group and following the first treatment session in the continued rehabilitation group.

Psychosocial risk profiles will be measured with the Örebro Musculoskeletal Pain Screening Questionnaire (ÖMPSQ) [37-40]. The 21 items of this questionnaire evaluate function, pain, psychological factors and fear avoidance on 11-point scales, yielding a total score range of 0–210. Higher scores indicate a higher risk of developing long-term problems.

Fear-avoidance beliefs will be measured with the Fear-Avoidance Beliefs Questionnaire (FABQ) [41,42]. This instrument measures both fear-avoidance beliefs about work (FABW) (11 items) and about physical activity (FABPA) (5 items) using seven-point scales. Sum-scores will be calculated for FABW (7 items, range 0–42) and for FABPA (4 items, range 0–24).

Pain coping will be assessed with the Pain Coping Inventory (PCI) [43]. The PCI comprises 33 items, evaluating three active and three passive pain coping dimensions on a four-point scale ranging from ‘seldom or never’ to ‘very often’.

### Blinding

Neurosurgeons will be blinded for group allocation, as randomisation and group allocation is performed after their involvement in participant recruitment. The research nurses will inform participants on treatment allocation and can, therefore, not be blinded. The nature of the postoperative strategies precludes blinding of the patient and the therapist. Besides, all measures are patient reported outcomes, and therefore outcome assessment is not blinded. The unequal size of intervention and control group, necessary for multilevel analysis, prevents blinding of the researcher and the statistician involved in outcome analyses.

### Sample size

Power calculations were based on a Cochrane review assessing the effectiveness of rehabilitation following lumbar disc surgery [18] and were performed for the three main outcomes (for all: power 0.9; alpha 0.05). To detect a clinical relevant mean difference in a multi-level analysis between the continued rehabilitation group and the watchful waiting group of 8 points on the ODI (SD 15), a total of 165 patients is needed. To detect a difference of 2 points (SD 3) for pain (11-point NRS) a total of 105 patients is needed. For detecting a 20% difference on the dichotomised global perceived recovery (recovered vs not recovered) a total of 150 patients is needed. Anticipating 15% potential study withdrawal, a total of 200 patients will be recruited. Participants will be unequally distributed (109 intervention vs. 91 control) to allow for multilevel analysis, taking into account the multilevel structure of the data in the intervention group.

### Data analysis

Baseline characteristics in both arms will be compared to check if randomisation has resulted in an equal distribution of the main outcome measures, prognostic factors and known confounding factors such as age, gender, living status and educational level.

The primary analysis will be an intention-to-treat analysis. All continuous responses will be analysed in a linear mixed model with responses at baseline and 3, 6, 9, 12 and 26 weeks. If necessary, missing items within a questionnaire will be imputed using multiple imputation techniques. In these analyses we will take into account the levels of neurosurgeon, primary care therapists, patient and time of measurement. The effect of interest for the present study will be the time by treatment interaction. Therefore, regression coefficients with 95% confidence intervals (CI) between baseline and follow up measurements will be calculated. If appropriate, analyses will be adjusted for patient characteristics that differ between the two groups. For the dichotomous outcomes we will use a generalised mixed model (logit link) with the same multilevel structure, and the effect of interest will be the difference between groups at each time point. Odds ratios with 95% CI between intervention and control groups will be calculated.

A per protocol analysis will be performed to estimate the extent to which protocol deviations may bias the results. A protocol deviation is defined as receiving one or more sessions of exercise therapy in the first six to eight weeks after surgery in the control group, or not receiving any sessions of exercise therapy in the first six to eight weeks postoperatively by participants in the intervention group.

### Economic evaluation

The economic evaluation will be performed according to the intention-to-treat principle and from a societal perspective. Multiple imputation according to the MICE algorithm will be used to impute missing cost and effect data [44].

All costs will be summed for each individual patient. Incremental cost-effectiveness ratios (ICERs) will be calculated by dividing the difference in total costs between the two groups by the difference in effects. Bias-corrected and accelerated bootstrapping with 5000 replications will be done to estimate the uncertainty surrounding the ICERs. Bootstrapped incremental cost-effect pairs will be plotted on cost-effectiveness planes to graphically illustrate the uncertainty around the cost-effectiveness ratios. A summary measure of the joint uncertainty of costs and effects will be presented using cost-effectiveness acceptability curves, which indicate the probability that the intervention is cost-effective in comparison with control at different ceiling ratios (i.e. the maximum amount of money society is willing to pay to gain one extra unit of effect). Sensitivity analysis on the most important cost drivers will be performed in order to assess the robustness of the results.

## Discussion

A systematic review that summarised results of several randomised controlled trials yielded moderate to low quality evidence for effectiveness of postoperative exercise programmes starting 4–6 weeks after first time lumbar disc surgery [18]. Exercise programmes seem to be more beneficial than no treatment, and high intensity exercises may be more effective than low intensity exercises. Little is known about the effectiveness of immediate postoperative interventions in these patients [18]. Besides, knowledge about cost-effectiveness of postoperative management is scarce [19]. Therefore, the REALISE trial aims to evaluate the effectiveness of an exercise therapy intervention starting in the first postoperative week, compared to a watchful waiting policy during the first six to eight weeks after surgery. To that end we mainly focus on the question whether exercise treatment leads to a faster recovery as compared to watchful waiting during this immediate post operative period. Furthermore, cost-effectiveness will be assessed by means of an economic evaluation. Treatment in the intervention group is based on a protocol that allows therapists to provide a tailored intervention. Also, both physiotherapists and exercise therapist will deliver the treatment, as this reflects current practice in the Netherlands. This will result in a variation across treatments and will improve generalisability of the findings. The results of this trial may lead to a more consistent postoperative strategy for patients who underwent surgery for lumbar disc herniation.

## Competing interests

The authors declare that they have no competing interests.

## Authors’ contributions

MvT, WP and RO conceived of the study idea and TO, MvT, WP, JB, RO participated in the development of its design. TO, CVL, LS, MA and RO developed the study protocols. TO drafted the manuscript. MvT, WP, JB, and RO revised the manuscript critically for important intellectual content. All authors read and approved the final manuscript.

## Pre-publication history

The pre-publication history for this paper can be accessed here:

http://www.biomedcentral.com/1471-2474/14/124/prepub
